# Apolipoproteins, as the carrier proteins for lipids, are involved in the development of breast cancer

**DOI:** 10.1007/s12094-020-02354-2

**Published:** 2020-04-18

**Authors:** Y. Zhou, G. Luo

**Affiliations:** Comprehensive Laboratory, Changzhou Key Lab of Individualized Diagnosis and Treatment Associated with High Technology Research, The Third Affiliated Hospital of Soochow University, Changzhou, 213003 China

**Keywords:** Breast, Carcinoma, Apolipoprotein D, Apolipoprotein E, Treatment

## Abstract

Apolipoproteins, the key components of lipoproteins, play vital roles in the combination and transportation of lipids. Numerous research articles have accumulated solid evidence that lipoproteins are closely related to various types of tumorigenesis. In this review, we focused on the associations between several apolipoproteins and breast carcinoma and distinguished the effects and significance of apolipoproteins in different locations to validate their roles in breast carcinoma development. For example, apoD and apoE in serum are viewed as risk factors for breast carcinoma. ApoD, apoE and apoA-I in mammary tissues inhibit tumor growth. Moreover, apoB, apoJ and apoA-I have the potential to function as diagnostic or prognostic markers in the clinic. ApoEdp and apoJ treatment on breast carcinoma could significantly restrict tumor growth. In general, the aim of this review was to further analyze the associations between some members of the apolipoprotein family and breast cancer.

## Introduction

Apolipoproteins are named for their association with lipids and their roles in lipid transport. Plasma apolipoproteins bind to the surface of lipoproteins and fulfill multiple functions in lipid metabolism [1], such as stabilizing the lipoprotein particle structure and serving as cofactors for enzymes. Apolipoproteins also participate in targeting lipoprotein particles as ligands for receptors in the cell [2]. As mentioned above, apolipoproteins are the protein component of high-density lipoproteins (HDL), low-density lipoproteins (LDL), very low-density lipoproteins (VLDL), intermediate density lipoproteins (IDL) and lipoproteins (a) [2, 3], which precisely explains how lipids and lipoproteins are transported and redistributed among various tissues. For example, apolipoprotein B (apoB) binds to LDL, VLDL, and IDL but not to HDL. The other members mostly bind to the surface of HDL, such as apolipoprotein A (apoA), apoB, apolipoprotein C (apoC) and apolipoprotein M (apoM) [4]. A multiple regression analysis suggested that the concentrations of apoA-I, apoA-II, apoB and apoE are all associated with LDL particle sizes [5]. The associations among HDL, LDL and various diseases [6, 7] have been widely acknowledged, which consequently suggests the participation of apolipoproteins in diverse diseases.

In recent decades, together with the improved standards of living and increased life expectancies, epidemic studies have reported associations among obesity, lipoproteins and malignancy. One report conducted by the International Agency for Research on Cancer (IARC) Working Group on Body Fatness in 2016 suggested that body fat is sufficiently linked to risks of more than a dozen cancers [8, 9]. Obesity mainly alters the metabolism of sex hormones, adipokine pathways, inflammatory factor pathways and other patterns of endocrine metabolism, which is consistent with the conclusion in the report described above. Since cholesterol is mainly transported by LDL and HDL, clinical trials have assessed the associations between lipid profiles and tumors, and they concluded that the levels of LDL cholesterol (LDL-C) affect the prognosis of carcinoma progression [10]. In contrast, HDL cholesterol (HDL-C) plays an inverse role in carcinogenesis by regulating cell cycle entry and cell apoptosis via the mitogen activated protein kinase-dependent (MAPK) pathway [11]. Furthermore, understanding the involvement and mechanism of apolipoproteins in carcinomas has subsequently become a popular research topic. According to research from Pearlman et al., apoD is the first apolipoprotein demonstrated to be associated with cancer. In the proliferative process of prostate cancer cells, stimulating the secretion of apoD contributes to the inhibition of cancer cell proliferation [12]. The significant roles of apoE genotypes and apoJ in prostate carcinoma development have also been confirmed [13, 14]. In the present review, we focused on the mechanisms of the effect of apolipoproteins, as the carrier proteins for lipids, on breast cancer (BC) development.

In 2018, there were more than 2 million new BC cases and ~ 0.63 million BC-related deaths worldwide. In Asia, BC incidence reaches 0.9 million, accounting for 43.6% of the total new cases worldwide, and the mortality is approximately 0.31 million, accounting for 49.6% of the total global BC-related deaths [15]. A hierarchical cluster analysis identified six molecular subtypes of BC on the basis of gene expression, including luminal subtype A, luminal subtype B, luminal subtype C, normal breast-like subtype, basal-like subtype, and ERBB2 + subtype. The proteins associated with BC are consistent with their genetic subclass, such as the estrogen receptor (ER), progesterone receptor (PR) and human epidermal growth factor receptor 2 (HER2), which may also be related to prognosis and survival rates. For instance, patients with BC lacking the amplification of the ER and HER2 genes tend to have poor survival rates. The triple negative tumor (TNT/TNBC) is one type of BC that does not express the three aforementioned protein receptors. A previous study demonstrated that one patient suffering from TNT had poor prognosis and high rate of metastasis [16]. Besides, menopausal state is another factor that closely associated with BC prognosis. Postmenopausal women are more likely to get breast cancer and have more aggressive forms of the disease, possibly because menopause throws hormones out of whack. Menopause also increases the occurrence of metabolic syndrome (MS) and type II diabetes (T2D) [17]. Consequently, the menopause state, MS, T2D and obesity are closely related with each other and affect BC development [18]. Breast tissue is mainly composed of the lobule of the mammary gland and adipose tissue. The roles of obesity and lipoproteins in BC have been illustrated in a substantial number of studies, but the conclusions are divergent. Taking lipoproteins as an example, two prospective studies, including approximately 5000 and 15,000 participants, identified a non-relationship between LDL-C or HDL-C and BC risk in 2016. Interestingly, the results from two other meta-analyses showed the opposite results for the relationships between HDL-C and BC risk among different menopausal females. In addition, the two meta-analyses both confirmed that LDL-C is not associated with BC risk. In 2018, however, one study reported positive connections between lipoprotein cholesterol and ER-positive BC [19]. The roles of lipoproteins in BC seem to be clear, but the roles of apolipoproteins in BC require more thorough investigation. In 1994, apoD was first proposed to distinguish subtypes of BC when combined with other prognostic factors [20]. In 2017, low levels of plasma apoA-I were indicated to independently predict the outcomes of patients with invasive ductal BC [21]. To elucidate the complexity of the apolipoprotein family members and their potential clinical uses, the present review aimed to identify the different roles of apolipoproteins in serum and in cancer tissues.

## ApoD and BC

**Fig. 1:**
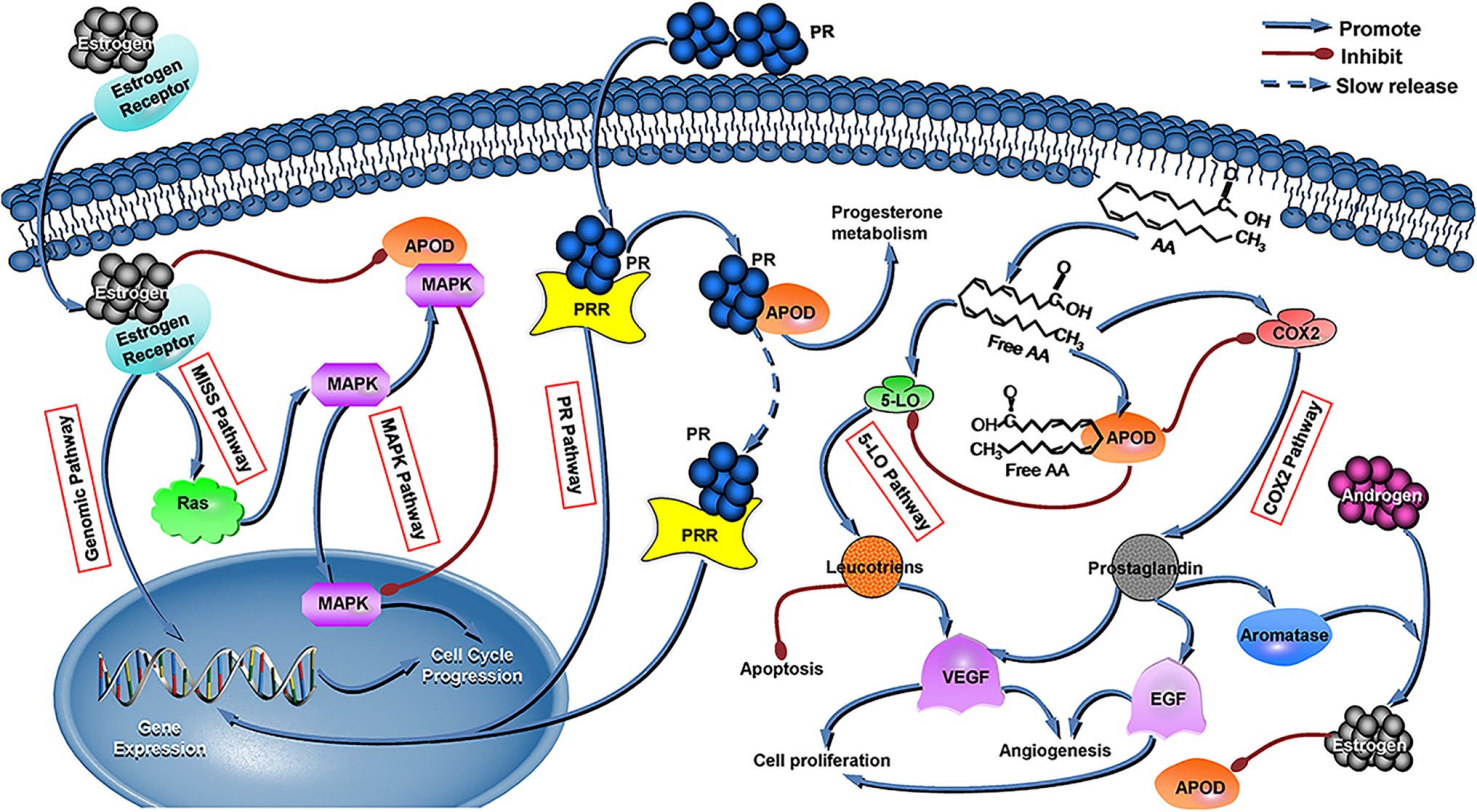
Potential mechanisms of ApoD acting on breast carcinoma cells

ApoD, also known as gross cystic disease fluid protein-24 (GCDFP-24) and progesterone-binding cyst protein (PBCP), is a member of the lipocalin superfamily. In 1963, human apoD was detected in plasma. Ten years later, McConathy et al. originally differentiated apoD from plasma HDL [22]. The apoD gene, which is located on the 3q26.2-qter chromosome, contains five exons, and its promoter region is in exon 1, where a number of response elements reside [23]. When combining other factors in plasma, the apoD-centered complex is active in catabolism by excreting cholesterol from peripheral tissues and transporting cholesteryl esters to the liver [24]. In addition to being present in the liver and intestine like other apolipoproteins, apoD has also been observed in the suprarenal gland, brain, endometrium, and certain connective tissues [25]. Three response elements concerning estrogen receptors have been discovered in the promoter region, and these elements exert strong inhibitory influences on apoD gene transcription [23]. As Simard et al. [26] demonstrated in human BC ZR-75-1 and MCF-7 cells, estrogen inhibits the secretion of apoD, and androgens exert the opposite effect. Another study performed by Simard et al. initially proved that physiological estrogen E_2_ concentrations distinctly reduce apoD gene expression and excretion, which inversely induces the proliferation of ZR-75-1 BC cells at the molecular level [27]. In addition, tamoxifen increases apoD concentration, and progesterone has bidirectional regulatory effects on apoD levels [28]. All of these factors that regulate apoD expression directly or indirectly affect the roles of this protein in BC development. Among the apolipoproteins, apoD was the first to be demonstrated to play a significant role in this carcinoma. Patients with mammary cysts tend to increase the risk of developing BC by at least threefold compared with that in normal females. ApoD is the primary cyst fluid component, and its plasma concentration in a mammary cyst patient is 1000 times higher than that in a healthy woman, which in turn indicates the role of apoD in BC carcinogenesis [29]. As early as 1977, GCDFP-24 was detected in the plasma of patients with BC, and the concentrations in females with advanced BC were frequently found to be > 150 ng/ml (normal range is 6–14 mg/dl) [30]. Later, in 1987, researchers also discovered that PBCPs had the potential to mark the development and metastasis of early carcinomas, suggesting that PBCPs may be novel prognostic indicators [31]. In 2001, the results from immunoperoxidase staining showed that 63.2% of tumors from 163 BC patients were positive for apoD immunostaining and that approximately 40.0% of tumors were strongly positive, with the remaining being moderately or weakly positive [20]. With the increased degree of malignancy of BC and the occurrence of metastasis, the apoD content decreases. Therefore, the presence of apoD is at its highest in benign tumors, whereas its expression is at the lowest level in invasive cancer and BC with metastasis [31]. Among BC patients, elderly and menopausal patients express higher apoD levels than those who are younger or who have not yet experienced menopause [20]. The above effects of apoD in BC may be attributed to its interactions with several critical pathways, including the ER, MAPK, PR, COX-2 and 5-LO pathways. First, one mode of ER signaling is the genomic pathway, which promotes gene expression in the nucleus and advances cell progression. The other mode of ER signaling is ER membrane-initiated steroid signaling (MISS-ER), which interacts with MAPK signaling via the rat sarcoma virus oncogene (Ras). A previous study validated the high mRNA level of MAPK expression in BC [32], and it demonstrated that apoD acts together with the MAPK signaling pathway. When apoD abundantly exists in the cytoplasm, it blocks the translocation of MAPK from the cytosol into the nucleus and hinders cell cycle progression, which consequently restricts cell proliferation [33]. However, as mentioned before, ER may inhibit the abundancy of apoD. Thus, the high expression of apoD, low expression of ER, and weak activity of MAPK all contribute to the low proliferation of cancer cells, which indicates a better prognosis for BC patients. Second, apoD binds progesterone with a strong affinity. By participating in the PR pathway partly by combining to and transporting progesterone, apoD inactivates PR metabolism. On the other hand, apoD may produce a “slow release” effect on progesterone and extend its gene expression in cells, which also hinders the proliferation of BC cells. Third, apoD interferes with the pathways of prostaglandin-producing cyclo-oxygenase-2 (COX-2) and 5-lipoxygenase (5-LO) enzymes by strongly binding to arachidonic acid (AA) [34, 35]. Redundant free AA, the substrate for COX-2 and 5-LO, is harmful to normal cells, because it promotes the COX-2 and 5-LO pathways. The end products of the two pathways are prostaglandins and leukotrienes, which stimulate cell proliferation, inhibit apoptosis and facilitate neo-angiogenesis. In addition, the activation of COX-2 in tumors is related to enhancement of cell invasiveness and motility. When apoD closely combines with free AA, such end products are reduced, and the malignant transformations caused by these products are subsequently avoided. Therefore, a low concentration of apoD and high expression of AA, COX-2 and 5-LO all lead to poorer BC survival rates. It is worth mentioning that prostaglandins are capable of inducing aromatase p-450 to convert androgens into estrogens [28], which continuously decreases apoD expression. In summary, apoD is involved in these signaling pathways, and such pathways interact with each other (Fig. 1).

ER pathways: Estrogen and its receptor promote gene expression and BC cell progression in the nucleus through the genomic pathway. The MISS-ER pathway promotes the cell cycle by interacting with the MAPK pathway via Ras. MAPK pathway: MAPK translocation from the cytosol into the nucleus enhances cell cycle progression and cell proliferation. Abundant apoD in the cytoplasm binds with MAPK and blocks its translocation, which consequently has an inhibitory effect on BC cell proliferation. ApoD is the second factor that combines ER signaling and the MAPK pathway due to the reduced apoD expression induced by ER. PR pathway: PR and the PR receptor (PRR) exert the same influence as ER on gene expression and cell proliferation. When apoD binds to progesterone with a strong affinity, some PR is allocated to the inactivation of metabolism. On the other hand, apoD may produce a “slow release” effect on PR and suppress gene expression in the nucleus, which also hinders the proliferation of BC cells. COX-2 and 5-LO pathways: Free AA promotes the COX-2 and 5-LO pathways. Prostaglandins and leukotrienes are the end products of the two pathways, and they inhibit apoptosis, facilitate neo-angiogenesis and stimulate cell proliferation. Prostaglandins not only promote angiogenesis but also induce aromatase, which converts androgens into estrogens. When apoD closely chelates with free AA, the downstream products are reduced, and the malignant reactions are subsequently avoided. ER decreases apoD expression, and the final effects of apoD on BC cells may depend on the expression or binding capability with the above cytokines.

There is an interesting saying that “apoD is a good citizen but a bad neighbor”, which may be attributed to apoD symbolizing cell senescence. When apoD is present in cancer cells, it promotes the senescence of tumor cells and inhibits cell proliferation. Upon apoD becoming abundantly present in stromal cells, the senescence of these stromal cells is not a good sign due to the decrease in something that could otherwise restrain the invasion of tumor cells, such as tissue inhibitors of metalloproteinases and serine [36]. In addition, senescent stroma produces a substantial amount of specific matrix metalloproteinases, epidermal growth factors and inflammatory cytokines, which subsequently negatively influence the prognosis of patients with BC [28, 37]. Therefore, the impact of apoD on cancerous epithelial cells and adjacent stroma is completely different [28]. Generally, treatment with tamoxifen inhibits ER expression and consequently decreases the proliferation of cells. However, the existence of apoD and ER is diverse. Treatment of apoD-negative and ER-positive BC patients with adjuvant tamoxifen not only increases the 10-year survival rates but also decreases the recurrence rate of this disease [38]. For apoD-positive and ER-positive BC patients, ER generally stimulates cell proliferation and inhibits apoD transcription. When tamoxifen is administered, it decreases the depressing functions of ER and upregulates apoD transcription. However, the new complex chelated from apoD and tamoxifen may affect the roles of tamoxifen in cell multiplication, which indicates that the treatment of apoD-positive BC may need to be changed or improved [28, 39]. In conclusion, apoD in plasma is associated with BC risk, and it may function as a predictor during tamoxifen treatment in BC. ApoD in cancer cells may inhibit cell proliferation and be beneficial to patient prognosis, while apoD in stromal cells has the opposite effect on cancer cells and may be unfavorable to patient prognosis.

## ApoE and BC

ApoE is located on chromosome 19q13.2 and consists of four exons that lie on the long arm of chromosome 19. On the chromosome, the apoE gene encodes three common alleles, including ε2, ɛ3 and ɛ4. The liver synthesizes approximately three-quarters of plasma apoE. ApoE is also secreted by numerous other tissues, such as the kidneys, ovaries, testes, skin and macrophages [40]. A liver X receptor (LXR) response element (LXRE) also regulates apoE expression. In addition to participating in transporting cholesterol and metabolizing lipids, apoE also has roles in restoring injured tissues, reacting to immunological regulation, and differentiating cell growth [41, 42]. Furthermore, apoE may also inhibit cancer cell proliferation and angiogenesis due to its high affinity for heparin and proteoglycans [41, 43]. However, the function of apoE in tumor cells is unclear as it has opposing effects in different cancers. In lung and ovarian carcinomas, apoE is a promoter for the growth and migration of tumor cells, but in melanoma, it is an inhibitory factor for angiogenesis and metastasis [44–46]. One study suggested that the gene expression regulated via LXR is relevant to the grades and characteristics of tumors [47]. Consequently, apoE is associated with tumor grades and properties by the mediator LXR. In addition, the receptor-binding region of apoE, especially the heparin-binding domain located between residues 142 and 147, critically affects its bioactivity. This specific domain mediates the adhesion of apoE to cellular heparin sulfate proteoglycan (HSPG), which is the holonomic component of the extracellular matrix on the cell surface and is widespread in nature. The competition by apoE and several viruses to combine with HSPG suggests that apoE is capable of anti-infection activity [48]. On endotheliocytes, HSPG generally presents as a basic fibroblast growth factor (bFGF) to its receptor, thus stimulating mitogenesis, promoting cell motility, and inducing angiogenesis. ApoE in endothelial cells competes with bFGF to bind HSPG, suggesting that apoE has anti-proliferative activity. Furthermore, apoE hinders tumor cell multiplications by inhibiting the interactions among heparin-dependent cell matrixes [43]. It has been reported that HSPG molecules bind to vascular endothelial growth factor (VEGF) and their receptors (VEGFR), significantly interfering with or inhibiting several cancer biology process, such as angiogenesis, cancer progression and tumor metastasis [49, 50]. However, whether the adhesion of apoE to HSPG affects these aspects of tumor biology induced by HSPG or not still requires additional investigation. In contrast, the roles of apoE secreted by cancer cells in the melanoma carcinoma model are not the same. Endogenic apoE combines with LRP1 and LRP8 receptors in cancer cells and endothelial cells, respectively, which not only hinders tumor cell invasion and metastasis but also restricts the recruitment of epithelium. These specific combinations inhibit metastasis and angiogenesis. However, high expression of a microRNA cluster in melanoma carcinoma, including microRNA-199-5p, microRNA199a-3p and microRNA-1908, restrains apoE expression. Consequently, the anti-metastasis and anti-angiogenesis effects induced by apoE are both blocked [46]. The roles of apoE are different among various cancer tissues due to the regulation induced by upstream molecules. The association of apoE with BC and how it impacts this carcinoma have been the focus of an increasing number of studies, but the findings still need further development and improvement. The findings regarding the associations between apoE and BC were not consistent at the beginning of the twentieth century. In Finland and Italy, Niemi M et al. and Zunarelli et al. clearly demonstrated that there are no statistically significant associations between the presence of apoE alleles and occurrence of this carcinoma [51, 52]. Moreover, a case–control study conducted by Kirsten et al. reported that patients in New York with one or two apoE4 genes and higher serum triglyceride levels may suffer from a quadrupled risk of developing BC [53]. In Taiwan, the presence of the ɛ2 allele could result in a tendency to develop BC in the left breast among those who have not yet experienced menopause [52]. As a result, the possible risks of experiencing BC may depend on differences in race. Subsequently, Tirtsa et al. attributed such inconsistent associations between apoE genotypes and BC risk to not considering tumor staging [54]. In their research, these authors specifically focused on patients with early-onset BC in Puerto Rico, including those aged 50 years and younger. These researchers found that the frequency of the apoE3/apoE4 allele is twice as common in women ≤ 50 years of age compared with that in normal women and that the odds ratio of apoE4 frequency among these females between non-BC patients and BC patients is 2.15. Furthermore, apoE4 levels are suggested to decrease tumor size among younger females [54]. Tirtsa et al. investigated how apoE4 acts on various stages of BC, particularly early-onset BC, and how apoE assists in predicting tumor size. In 2015, another analysis demonstrated that the posttranslational modifications of apoE, such as methylation and dihydroxylation, play roles in tumor development [55]. As Xu et al. reported that serum apoE levels are notably upregulated in patients with BC and that this upregulation is associated with the tumor-node-metastasis stage. Among the apoE-positive groups, those with a higher apoE level tend to exhibit a poorer prognosis, and univariate and multivariable COX regression analyses showed that apoE in plasma could be independently considered as a prognostic risk factor [56]. In addition, apoEdp (Ac-LRKLRKRLLLRKLRKRLL-amide), a tandem-repeat dimer peptide derived from the apoE receptor-binding region between residues 141 and 149, has shown anti-infection properties in vivo and vitro. Partha et al. first demonstrated the inhibitory effects of apoEdp on tumor growth and angiogenesis in 2011. ApoEdp hinders the existence, multiplication, migration, and capillary formation of endothelial cells, and apoEdp treatment significantly restricts tumor growth in nude mice. These studies indicated that apoEdp might have the potential to function as an agent for the suppression of tumor development in the clinic. In summary, serum apoE has a positive association with BC malignancy degree, while apoE expression in BC tissues may have a negative correlation with BC development. The definite mechanisms for these findings urgently need to be clarified, including the identification of upstream microRNAs that inhibit endogenous apoE in BC tissues as in melanoma cancer [46], which may be a major breakthrough in the research of apoE and BC.

## ApoA-I, ApoJ and BC

Apolipoprotein A-I, a subtype of apoA, is a cofactor of LACT and plays a role in reverse transporting cholesterol from peripheral tissues to the liver. The liver and small intestine synthesize this subtype of apolipoprotein [57]. Compared with other apolipoproteins, apoA-I is the most abundant in HDL particles [58]. In addition to releasing cholesterol from cells, apoA-I participates in several physiological or pathological processes of anti-inflammation, anti-oxidation and anti-apoptosis [59]. In the process of tumor occurrence and development, apoA-I has been reported to significantly inhibit the growth and invasion of tumor cells [60]. In addition, several studies have found that in multiple xenograft mice models, HDL that contains human apoA-I effectively inhibits the activities induced by tumors in ovarian carcinoma, malignant melanoma, and Lewis lung cancer [61, 62]. When focusing on the proliferation of BC cells, these studies assessed the polymorphisms of apoA-I that are associated with BC in great detail and even discussed its effect on metastasis of this tumor. For example, the variations of the apoA-I gene (A1-75 G/A and + 83 C/T) distinguish individuals with a higher risk of developing BC, indicating that lower apoA-I expression is associated with higher BC risk [63]. Another prospective study also demonstrated that apoA-I in serum is inversely associated with risk of BC [64]. Furthermore, multivariable regression analysis suggested that increasing apoA-I levels is closely associated with a decreasing risk of invasive ductal carcinoma (IDC) and that low apoA-I could independently predict the poor prognosis of patients with this disease. Upon diagnosing IDC, the apoA-I level is inversely associated with tumor size [21]. Liu et al. demonstrated that apoA-I is of higher reliability in distinguishing whether patients with BC have intraocular metastases, which provides direct evidence to support the claim that apoA-I is involved in BC metastasis [65]. As we described above, higher apoA-I levels in plasma tend to decrease BC risk. However, the overexpression of apoA-I in serum may not absolutely benefit BC patients. First, redundant apoA-I increases 27-hydroxycholesterol (27-HC) expression, which generally advances breast tumor growth [62]. Second, the reduction in oxidized LDL caused by the overexpression of apoA-I equal to the increase in the LDL level promotes multiplication and migration in ER-negative BC cells, and oxidized LDL may promote MCF-7 cell proliferation [66, 67]. Interestingly, Lídia et al. confirmed the role of apoA-I in mammary tissues through a mouse model with inherited BC. After administrating one specific apoA-I mimetic peptide (D-4F) in the BC mouse model, they found that although D-4F does not decrease 27-HC expression in breast tissue, it inhibits oxidized LDL levels in plasma and prevents the proliferation of BC MCF-7 cells [62]. Overall, these articles provided sufficient information about apoA-I and BC from the occurrence to distant metastasis and explored the effects of apoA-I expression in serum and mammary tissues for BC. In addition, the conclusions of these studies are widely accepted. However, as the number of these studies is fewer compared to those on apoD, these conclusions still need to be further verified.

ApoJ, known as clusterin, is involved in lipid transport, cell adhesion, programmed cell death and complement cascade reaction. The major actions induced by apoJ prevent cell death. In carcinoma development, apoJ inhibits the cancer cell apoptosis and then has effects on tumor growth and metastasis [14]. Recently, many studies have focused on how apoJ affects BC development. The presence of ApoJ is correlated with the status of negative estrogen and progesterone receptor. Among TNBC patients, the overexpression of apoJ accounts for higher percentage. Furthermore, knocking down and silencing apoJ not only depresses BC cell proliferation but also decreases cell transfer and invasion capability of MDA-MB-231 cells [68, 69], which may offer a novel and significant therapeutic method for BC.

## ApoB, ApoC-I, ApoM and BC

As previously mentioned, the apolipoprotein family contains numerous other members, such as apoB, apoC and apoM. These apolipoproteins are not as closely associated with BC as apoD and apoE. Therefore, we will review the existing or potentially existing hallmarks of these apolipoproteins in the carcinogenesis and development of BC. First, the associations among apoB, apoC-I and BC will be described, which are supported by existing literature. Second, we will aim to provide a theoretical conjecture regarding the potential roles of apoM in BC.

ApoB transports lipids into cells in the human body [70], and the serum apoC-I level is positively correlated with HDL concentrations. Both apolipoproteins are involved in lipid metabolism and cancer biology [1, 71]. The investigation into the connections between apoB gene polymorphisms and BC risk reported by Liu et al. concluded that polymorphisms, such as 12,669 GA and 7673 CT, significantly increase the risk of this tumor, particularly in women undergoing menopause [72]. Once breast carcinoma develops intraocular metastasis, apoB functions as a risk factor like apoA-I [65]. Whether the immediate role of apoB in mammary tissues is similar to that of apoA-I needs to be determined in the future. Jennifer et al. conducted a follow-up analysis and found that the ratio of apoB/apoA-I possesses a modest association with severe BC [73]. In addition, apoB may establish indirect effects on BC development via 27-hydroxycholesterol (27-HC) and LDL. Importantly, 27-HC is mainly transported by LDL and could stimulate ER-positive MCF-7 cells, and apoB is the major structural protein of LDL-C and determines LDL-C expression [19]. In 2010, another apolipoprotein family member, apoC-I, emerged as a novel marker in BC. When measuring the serum concentration of apoC-I, it was revealed that this protein exhibited low expression levels in the plasma of BC patients. In 2016, Sun et al. purified and identified the specific peptides of apoC-I through SDS-PAGE electrophoresis and matrix-assisted laser desorption/ionization time-of-flight mass spectroscopy (MALDI-TOF–MS). This peptide restrains the proliferation of MCF-7 and MDA-MB-231 cells and inhibits the growth of breast tumors in xenografted nude mice [71]. The conclusions confirmed by such experiments in vivo and in vitro were similar to those for apoEdp and D-4F, and they provided insight into the mechanisms in this inhibitory process. Another study focusing on apoC-I in TNT development has revealed the significant role of apoC-I in this malignant tumor. When diagnosing TNBC occurrence and development, apoC-I could be helpful to distinguish TNBC from non-TNBC cases [74]. The unique potential characteristics of apoC-I in TNBC are of high value in the early detection and evaluation of prognosis in TNBC. In summary, measurement of apoB and apoC-I in BC may be valuable to the clinic.

**Fig. 2:**
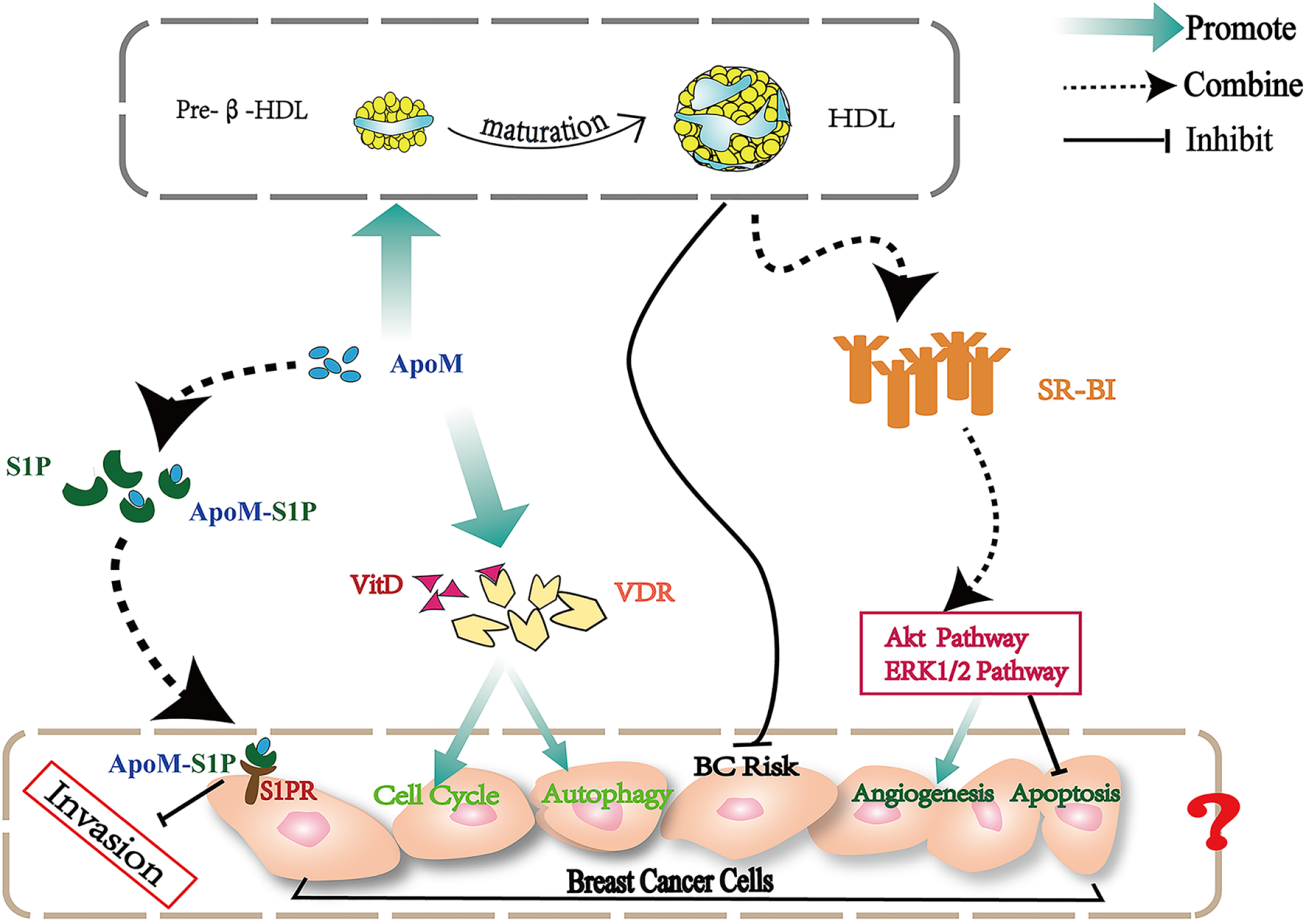
The potential associations between apoM and breast carcinoma cells

ApoM, isolated from chylomicrons, was first identified by Xu and Dahlback at the end of the twentieth century. ApoM is primarily bound to HDL particles and is present on low levels in LDL particles [4]. ApoM has been demonstrated to be involved in the development of different types of tumor, such as colorectal cancer and lung carcinoma [75, 76]. Recently, apoM has been identified as a carrier of sphingosine-1-phosphate (S1P), and it maintains the stability of the S1P concentration in plasma [77]. Since S1P inhibits the invasion of several tumor cells, apoM could be associated with certain types of cancer via S1P, which may be a novel line of research in future [77, 78]. In 2010 and 2012, Luo and Mu et al. revealed the involvement of apoM in colorectal cancer. When making comparisons to normal or benign colorectal tissues, the levels of apoM mRNA and protein are decreased in cancer tissues [79]. In contrast, colorectal cancer patients with lymphatic metastasis express significantly higher levels of apoM mRNA in colorectal tissues [76, 79]. In 2018, Zhu and Luo et al. clarified the role of apoM in non-small cell lung cancer (NSCLC) by increasing the regulation of S1P receptor 1, and they demonstrated that overexpression of apoM may promote NSCLC oncogenesis [75]. To the best of our knowledge, there is currently no research regarding apoM in BC. However, studies focusing on apoM in BC could have notable potential, as it can be hypothesized that apoM inhibits the proliferation of BC. Early in 1993, a study revealed that higher HDL-cholesterol could be a factor indicating a lower risk of BC [6]. The roles of HDL in mammary tumor tissues are inconsistent with the abundant existence of scavenger receptor class B type I (SR-BI), which is a receptor of HDL. High expression of SR-BI has been reported to be associated with tumor cell anti-apoptosis and angiogenesis via the Akt and ERK1/2 pathways. Pre-β-HDL is the immature form of HDL, and the maturation process cannot be separated from the effects of apoM [80]. As a result, the role of apoM in BC is of great significance. Another breakthrough in research confirmed that apoM is also involved in upregulating VDR expression [81]. Importantly, circulating levels of vitamin D contribute to protecting females against BC. In addition, Vitamin D and its receptor (VDR) modulate the autophagic process and cell death in BC cells and normal mammary tissue [82]. Subsequently, apoM exerts an inhibitory influence on BC development via increasing the VDR level. S1P is the third factor that links apoM and BC. S1P was demonstrated to suppress the motility of BC cells and hinder cell proliferation and growth of BC [78]. When apoM stimulates S1P, the upregulated S1P restrains BC development. In summary, apoM is a potential and emerging factor in the occurrence, development and prognosis of BC (Fig. 2).

ApoM carries S1P to bind with S1PR, which maintains the stability of the S1P concentration in plasma. S1P inhibits the invasion of breast tumor cells. Increased Vitamin D (Vit D) expression induced by apoM and its receptor (VDR) modulates the autophagic process and cell death in BC cells and normal mammary tissue. Furthermore, apoM promotes the maturation of HDL from pre-β-HDL. A high concentration of HDL-C lowers the risk of BC. However, the diverse existence of SR-BI, a receptor of HDL, in BC tissues may change BC development by affecting tumor cell anti-apoptosis and angiogenesis via the Akt and ERK1/2 pathways. Consequently, the roles of apoM in BC are worthy of exploration.

## Conclusions

Apolipoproteins bind to the surface of lipoproteins and have a role in transporting and metabolizing lipids. Various apolipoproteins form the diverse lipoproteins. These apolipoproteins are not only involved in coronary-artery disease, atherosclerosis, MS and T2D but also participate in various types of carcinoma. The present review discussed the roles of apolipoproteins in BC and summarized the different effects of apolipoproteins in this disease (Table 1). In terms of the chronological order of discovery, apoD and apoE are the earliest discovered among the apolipoproteins, followed by apoA-I, apoJ, apoB and apoC-I. The roles of apolipoproteins in serum and in cancer tissues are different. For example, apoD, apoA-I and apoB in plasma function as a risk factor for BC. Moderately high levels of apoA-I in plasma indicate a decreased BC risk, but apoA-I overexpression has the opposite influence. When these apolipoproteins, including apoD, apoE and apoA-I, are present in cancer tissues, they tend to inhibit tumor growth, and these apolipoproteins may be used in the clinic as therapeutic targets. Last, apoD, apoA-I and apoB are all involved in BC metastasis. ApoC-I peptide distinguishes TNBC from non-TNBC, and it may predict the prognosis of TNBC patients. The apoB/apoA-I ratio may indicate severe BC. Furthermore, apoEdp and apoJ treatment on BC could significantly restrict tumor growth. In the last part of the present review, we also hypothesized about the roles of apoM in BC, such as inhibiting the development of BC and predicting the prognosis of BC patients. However, such investigations are rare at present, and further research specifically on apoM would enrich the current knowledge of the associations between the members of the apolipoprotein family and BC.

**Table 1:** Effects of apolipoproteins in BC development

Apolipoproteins	Location	Effects and significance
ApoD	Serum	ApoD expression is positively associated with BC risk
	Cancer tissue	ApoD inhibits tumor cells proliferation ApoD expression is inversely associated with BC malignant degree and is considered as a prognostic factor
	Adjacent stroma	ApoD promotes stromal cells senescence and then advanced tumor invasion
ApoE	Serum	ApoE expression shows a positive association with the degree of BC malignancy
	Cancer tissue	ApoE may inhibit the formation of angiogenesis and the proliferation of BC cells
ApoA-I	Serum	ApoA-I expression is inversely associated with BC risk and tumor size Overexpression of apoA-I may not benefit BC patients ApoA-I is involved in BC metastasis
	Cancer tissue	Administration of apoA-I mimetic peptide (D-4F) in mammary tissues prevents the proliferation of BC MCF-7 cells
ApoJ	Cancer tissue	ApoJ inhibits the apoptosis of cancer cells, and its expression is positively correlated with ER- or PR- BC, especially TNBC Knocking down or silencing apoJ inhibits tumor growth and metastasis
ApoB	Serum	ApoB functions as a risk factor for BC metastasis; ApoB may promote ER + MCF-7 cell multiplication via 27-HC
ApoC-I	Serum	ApoC-I has low expression in BC patients ApoC-I distinguishes TNBC from non-TNBC and is valuable to detect and prognosticate TNBC development ApoC-I peptides suppress the proliferation of MCF-7 and MDA-MB-231 cells
ApoM	Cancer tissue	ApoM may inhibit the proliferation of breast carcinoma cells

## Data Availability

Not applicable.
